# Multiple organ failure leading to death after ingestion of *Caltha palustris*: A case report

**DOI:** 10.1097/MD.0000000000027891

**Published:** 2021-11-19

**Authors:** Keun Taek Lee, Won Young Sung

**Affiliations:** Department of Emergency Medicine, Daejeon Eulji Medical Center, Daejeon, Republic of Korea.

**Keywords:** bradycardia, gastroenteritis, plant poisoning, protein-losing enteropathies, shock

## Abstract

**Rationale:**

Studies have previously reported misidentifying *Caltha palustris (C. palustris)* as *Ligularia fischeri* and its subsequent ingestion leading to abdominal pain and gastrointestinal symptoms, which are alleviated immediately. Bradycardia and hypotension may persist for several days, and an infusion of dopamine can restore a healthy state without complications. We report a case of *C. palustris* poisoning with protein-losing enteropathy that has not been reported previously. The patient died of multiple organ failure, and exhibited more severe clinical deterioration than previous cases due to prolonged shock.

**Patient concerns:**

A 70-year-old woman was admitted to the emergency department (ED) with complaints of epigastric pain, vomiting, and diarrhea after ingestion of a poisonous plant presumed to be *C. palustris*. The patient presented with bradycardia and hypotension after ED admission, and vasopressor infusion improved bradycardia but not hypotension, while the patient complained of severe epigastric pain.

**Diagnoses:**

Abdominal computed tomography showed luminal distention and edematous thickening of the entire stomach lining, as well as small and large intestinal wall edema, indicating severe gastritis and enterocolitis. The laboratory test results suggested severe hypoalbuminemia, while the arterial blood gas analyses showed a continuous increase in metabolic acidosis.

**Interventions:**

As plant poisoning was suspected, activated charcoal was administered to the patient, followed by administration of vasopressors and other conservative therapies. Continuous renal replacement therapy (CRRT) was used for metabolic acidosis of increasing severity.

**Outcomes:**

Despite the administration of vasopressors and other conservative therapies, the state of shock persisted, and metabolic acidosis did not improve even after CRRT. Ultimately, the patient died of multiple organ failure.

**Lessons:**

For many poisonous wild plants, the precise profile of toxic compounds and mechanisms of action remain to be identified; when there is insufficient literature reporting on suspected plant poisoning, the medical personnel providing the treatment should consider the various side effects that differ from the reported ones and the possibility of more severe clinical progress and poor prognosis.

## 1 Introduction

Wild plants have long been used as a key food source in certain countries with food shortages, and they are also used as medicinal herbs to treat various diseases. If a nonexpert collects a wild plant, misidentification of the plant as an edible plant of a similar appearance occurs, and most patients visited to the emergency department (ED) who acquire plant poisoning through this route. In South Korea, *Caltha palustris* (*C. palustris*) is one of the most well-known poisonous plants that may cause plant poisoning as it is morphologically similar to *Ligularia fischeri* (*L. fischeri*).^[1]^ To date, *C. palustris* poisoning was previously reported once in 2 patients with presentations of transient abdominal pain and gastrointestinal symptoms; the associated bradycardia and hypotension were treated using a dopamine infusion that restored a healthy state after several days. No other complications occurred after conservative therapy.^[2]^ The present case report, however, describes a case of *C. palustris* poisoning with more severe clinical progress than previously reported, involving severe gastroenteritis and consequent protein-losing enteropathy. Hypotension persisted even after administration of vasopressors and other conservative therapies, and metabolic acidosis could not be treated using hemodialysis, resulting in patient death due to multiple organ failure.

## 2 Case presentation

**Figure 1:**
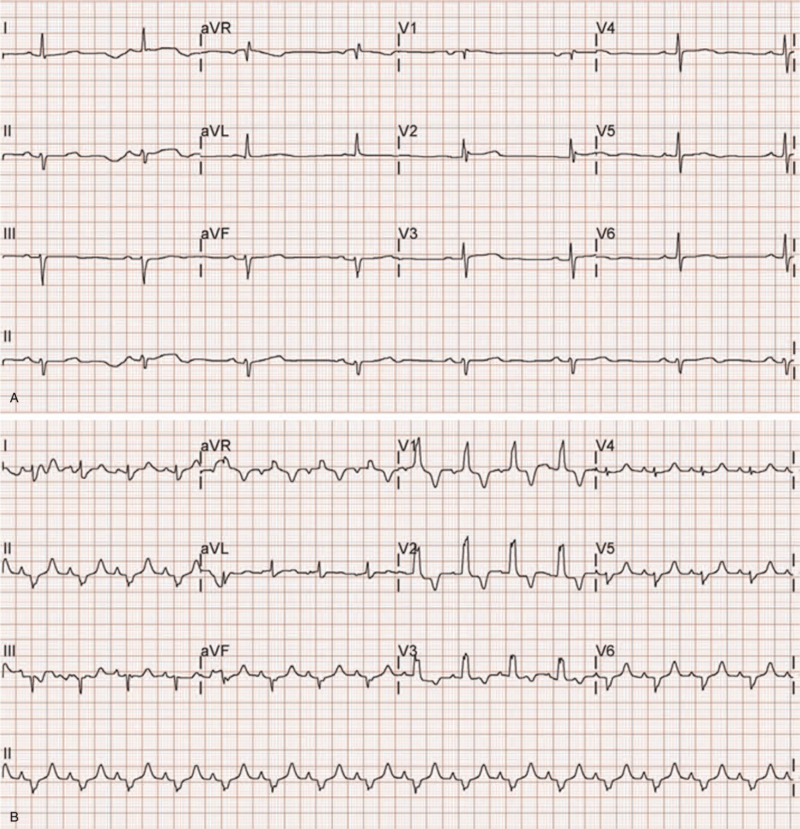
(A) The first electrocardiography performed after emergency department admission showed sinus bradycardia with a heart rate of 45 beats/min; (B) Following dopamine administration, the heart rate increased and a right bundle branch block was visible on the electrocardiography.

**Figure 2:**
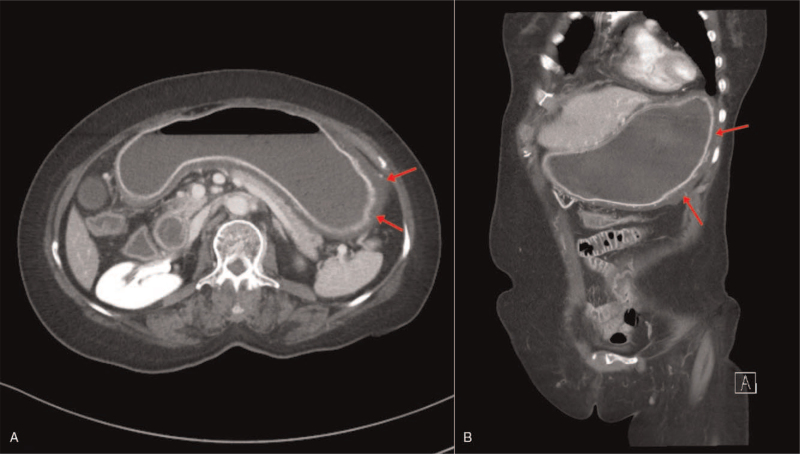
The first abdominal computed tomography performed at the emergency department showed luminal distention and wall thickening of the entire stomach (red arrows).

**Figure 3:**
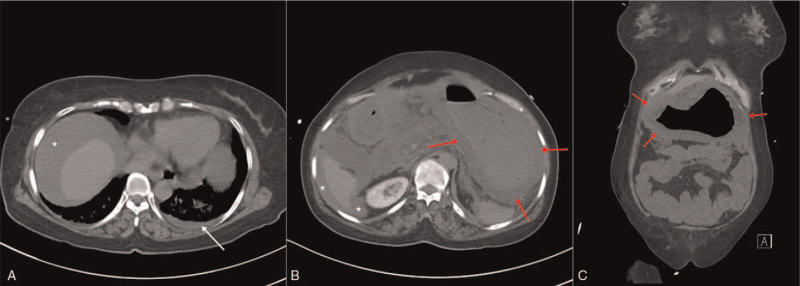
(A) The chest computed tomography showed ascites (white star) and a degree of pleural effusion on the left (white arrow); (B, C) The follow-up abdominal computed tomography showed more severe gastric wall thickening (red arrows) and ascites (white stars).

A 70-year-old woman with no notable medical history was admitted to the ED with complaints of epigastric pain for 2 hours prior to admission and over 10 bouts of vomiting and diarrhea. The patient reported having a shared lunch with her family 3.5 hours prior to the onset of symptoms. The lunch comprised a blanched mountain herb assumed to be *L. fischeri* given to her by an acquaintance as collected from the mountain. Other family members (daughter and grand-daughter) who ate with the patient also experienced abdominal pain, vomiting, and diarrhea, all of which disappeared by the time they visited the ED with the patient. Upon ED admission, the patient's vital signs were as follows: blood pressure, 120/70 mm Hg; pulse rate, 48 beats/min; respiratory rate, 18 breaths/min; and body temperature, 35°C. The patient had clear consciousness, and physical examination revealed severe epigastric tenderness. Initial electrocardiography (ECG) upon admission showed sinus bradycardia with a heart rate of 45 beats/min (Fig. 1A), for which continuous ECG monitoring was performed. The plain chest radiograph was unremarkable. A complete blood count at ED admission showed the following: White blood cells 12,530/μL; hemoglobin 16.2 g/dL; hematocrit 49.6%; and platelets 219,000/μL, while the results of chemical batteries, electrolyte test, C- reactive protein, and cardiac enzyme test were all normal. Arterial blood gas analysis (ABGA) results were pH 7.31, paO_2_ 79 mm Hg, paCO_2_ 38 mm Hg, HCO_3_^-^ 19.1 mEq/L, lactate 1.1 mg/L, and O_2_ saturation 98% (Table 1). During continuous ECG monitoring, a change in heart rate from as low as 43 beats/min to 66 beats/min was observed, although the blood pressure after 30 minutes of ED admission was 71/53 mm Hg, the patient did not show symptoms of shock, such as dizziness, and complained only of epigastric pain. As hypotension was detected, the patient was rapidly administered physiological saline, while for epigastric pain, a proton-pump inhibitor was administered. Given that the other family members who consumed the plant with the patient also showed similar symptoms, the patient was diagnosed with plant poisoning, and 50 g of medicinal charcoal (Heuk powder) was orally administered. The patient was then administered up to 1 L of physiological saline, after which continuous intravenous (IV) infusion of physiological saline was rapidly administered to the patient; however, severe fluctuations in blood pressure from 69/46 mm Hg at minimum and 140/84 mm Hg at maximum and a heart rate change from 45 beats/min at minimum to 74 beats/min at maximum, were observed. Two hours after ED admission, blood pressure and pulse rate continued to show severe fluctuations, and hypotension and bradycardia persisted. Thus, peripheral IV infusion of dopamine (10 μg/kg/min) was administered to the patient. Administration of dopamine led to the disappearance of bradycardia (Fig. 1B), but hypotension persisted. The dopamine dose was then increased to 20 μg/kg/min following the insertion of the central venous catheter (central venous pressure 11 CmH_2_O); since hypotension persisted, an infusion of norepinephrine was started. Subsequently, since the patient continued to complain of severe epigastric pain, an emergency abdominal computed tomography (CT) scan was performed which detected luminal distention, edematous thickening of the entire stomach wall, and small and large intestinal wall edema, suggesting severe gastritis and enterocolitis (Fig. 2). After 4 hours of ED admission, the ABGA showed pH 7.15, HCO_3_^-^ 10.1 mEq/L, and lactate 3.0 mg/L (Table 1), indicating an increase in the severity of metabolic acidosis. Therefore, sodium bicarbonate administration was given to the patient. Six hours after ED admission, the patient developed drowsiness with persistent hypotension. Despite the infusion of the maximum dose of norepinephrine, the systolic blood pressure remained at below 50 mm Hg; thus, an infusion of epinephrine was started. Despite the infusion of epinephrine, the blood pressure remained persistently low, and the patient began to show stupor with abdominal distension. The follow-up chemical batteries showed normal liver enzymes and creatinine level, although albumin and total protein levels had decreased to 2.0 g/dL and 4.0 g/dL, respectively. The follow-up ABGA also indicated a further increase in metabolic acidosis (Table 1). The patient developed mental change, and as O_2_ saturation steadily decreased despite external O_2_ supply, intubation and artificial ventilation were performed. The follow-up monitoring of albumin level indicated a further decrease to 1.0 g/dL (Table 1), abdominal distension increased in severity; thus, chest and abdominal CT were performed again. Chest CT revealed a small amount of pleural effusion on the left side, and the abdominal CT indicated an increase in the severity of edematous wall thickening of the stomach, with ascites in the abdominal cavity (Fig. 3). The patient was then administered an IV albumin to correct the reduced albumin level and abdominal ascites. As shock persisted despite conservative therapies with sodium bicarbonate and vasopressor injections, and as the level of lactic acid continued to increase on the follow-up ABGA with steadily increasing metabolic acidosis (Table 1), the patient was admitted to the intensive care unit after 10 hours of ED admission, and continuous renal replacement therapy (CRRT; CVVHDF mode) was performed. However, the chemical batteries after intensive care unit admission continued to show lower than normal levels of albumin at 2.8 g/dL and total protein at 3.6 g/dL, respectively. Despite CRRT, metabolic acidosis and hyperlactacidemia (lactate >15.0 g/dL) persisted (Table 1). The chemical batteries at hospital day 3 indicated an aspartate aminotransferase/alanine aminotransferase level of 3064/3314 IU/L and an increase in creatinine to 1.76 mg/dL, while the cardiac enzyme test showed CK-MB ≥300 ng/mL and an increase in troponin-T to 3.576 ng/mL, indicating a possibility of multiple organ failure. The patient died despite continued treatment.

**Table 1:** Changes in laboratory results over time after visiting the emergency department.

	Initial ED	ED 2 hr	ED 4 hr	ED 6 hr	Ed 8 hr	ED 10 hr (HD2)	ED 20 hr	ED 28 hr (HD3)
White blood cells (cell/ μL)	12,530	–	–	18,370	-	21,940	–	6040
Hb (g/dL)	16.2	–	–	19.7	-	17.7	–	14.3
Hematocrit (%)	49.6	–	–	60.3	-	55.1	–	43.9
Platelets (cell/ μL)	219,000	–	–	230,000	-	162,000	–	85,000
AST/ALT (IU/L)	35/24	–	–	32/14	39/10	93/56	–	3064/3314
Creatinine (mg/dL)	0.8	–	–	0.85	0.78	1.54	–	1.76
Albumin (g/dL)	5.0	–	–	2.0	1.0	2.8	–	2.8
Total -Protein (g/dL)	8.9	–	–	4.0	2.4	3.6	–	3.5
C-reactive protein (mg/dL)	0.06	–	–	0.35	0.35	0.61	–	0.77
CK-MB (ng/mL)	6.25	–	–	19.77	-	21.04	–	>300
Troponin-T (ng/mL)	0.015	–	–	0.023	-	0.196	–	3.570
ABGA								
pH	7.31	7.27	7.15	7.08	7.33	7.26	6.92	7.15
paO_2_ (mm Hg)	79	69	110	98	237	84	81	55
paCO_2_ (mm Hg)	38	31	29	26	33	26	40	35
HCO_3_^-^ (mEq/L)	19.1	14.2	10.1	7.7	17.4	14.5	8.2	12.2
Lactate (mg/L)	1.1	1.3	3.0	7.0	13.0	>15.0	>15.0	>15.0

ABGA = arterial blood gas analysis, ALT = alanine aminotransferase, AST = aspartate aminotransferase, CK-MB = creatine kinase MB, ED = emergency department, Hb = hemoglobin, HD = hospital day.

## 3 Discussion

Plant exposures remain in the top 25 reported exposures to US poison centers, and the identity of the plant is often unknown.^[3]^ It is rare to find a non-expert to accurately differentiate edible wild plants and poisonous plants with similar appearances or a patient to have accurate knowledge of the name of the plant he or she ingested. Medical personnel at the ED may frequently encounter patients with plant poisoning, despite not having sufficient knowledge of poisonous plants. Therefore, the identification of unknown toxic plants can pose challenges for medical personnel. For patients who bring the poisonous plant to the ED, the medical personnel may manually compare the plant with photos on the Internet or in relevant literature for primary identification, but this is an extremely difficult and time-consuming process, and relying on morphology alone, some plants may not be correctly identified.^[4]^ When such methods fail, plant identification may be requested from a botanist or the toxicity information center in the local region, but this is likely to consume a considerable amount of time to prevent any practical aid in treating the patient already showing the toxicity symptoms. For cases without any sample of the poisonous plant or with difficulty in identification, the plant may be identified in the reverse sequence from the name of the misidentified edible wild plant to search for the poisonous plant in the region with similar appearances and a history of frequent plant poisoning. Similarly, in this case, the patient did not report to the hospital with the ingested plant, and upon being instructed to bring the plant to the ED, the family of the patient reported that the plant had already been discarded after cooking. To accurately identify the poisonous plant for subsequent treatment, the family made contact with the acquaintance who had collected the plant in the first place, but only a photo of the blanched plant could be obtained to ensure accurate identification. However, the plant responsible for poisoning in the present case was conjectured to be *C. palustris* based on the following evidence: first, the acquaintance had referred to the plant as *L. fischeri* upon sharing it with the patient; second, the toxidrome (abdominal pain, bradycardia, and hypotension) of the patient initially showed similarities to the cases reported for the misidentification of *C. palustris* as *L. fischeri*.^[2]^

*C. palustris*, known as marsh marigold, is a small-to-medium-sized perennial herbaceous plant of the family Ranunculaceae, native to marshes, fens, ditches, and wet woodland in temperate regions of the Northern Hemisphere.^[5]^*C. palustris* contains biologically active substances, both primary and secondary. Its active principle comprises ranunculin, magnoflorine, and triterpene saponins.^[6]^ Many species of Ranunculaceae accumulate glycoside ranunculin in the vacuole. Upon hydrolysis, it is split into active protoanemonin, which can alkylate proteins. Protoanemonin is a potent vesicant that primarily irritates the mucus membranes of the digestive system, followed by severe inflammation.^[7]^ Large amounts of terpenoid saponins cause direct irritation of the digestive tract. In most instances, the disease is of only mild-to-moderate severity and occurs several hours after ingestion, with transient diarrhea and perhaps vomiting.^[8]^

In contrast to the previously reported cases of *C. palustris* poisoning, in which the patients presented with abdominal pain and gastrointestinal symptoms that lasted for 4 to 5 hours, followed by improvements, the patient in the present case complained of persistent epigastric pain after poisoning; despite the emergency treatment, the gastric wall edema increased with abdominal ascites and distension. The initially normal albumin and total protein levels at ED admission decreased suddenly, presumed to be due to the toxicity of protoanemonin and saponin of *C. palustris* causing mucosal injury and consequently serum protein loss via the gastrointestinal tract, that is, protein-losing enteropathy. In patients with protein-losing enteropathy, protein loss via the gastrointestinal mucosa will increase to almost 60% of the total albumin.^[9]^

In a previous case report, the authors claimed that the action of saponin in *C. palustris* caused bradycardia and hypotension.^[2]^ A mixture of triterpenoid saponin extract of *Aspidosperma fendleri* induced reductions in mean arterial blood pressure and heart rate, similar to those induced by propranolol in spontaneously hypertensive rats.^[10]^ The hypotensive effect of red ginseng is mainly due to saponin in rats, and this effect may be due to an increase in nitric oxide production.^[11]^ Hiwatashi et al^[12]^ showed that a triterpenoid saponin from soybean inhibited human renin activity in vitro improved hypertension in spontaneously hypertensive rats. Chen et al^[13]^ showed that saponins reduce systemic blood pressure and block the circulating and tissue renin-angiotensin-aldosterone system in spontaneously hypertensive rats. Magnoflorine was also found to exert hypotensive effects in several animals.^[14]^ These findings indicate that plants containing an abundance of saponin and magnoflorine compounds can cause cardiovascular symptoms, such as bradycardia and hypotension, upon ingestion.

To determine the reason for differences in severe clinical progress and prognosis between the previous cases and the present case, the time of ED admission and the time of first symptom were compared. All patients exhibited the first symptoms after 3.5 to 4 hours of ingestion of the poisonous plant, and the patients in previous cases were admitted to the ED after 7 hours of plant ingestion with hypotension and bradycardia, the patient in the present case was admitted after 5.5 hours; despite earlier admission, the patient showed more severe clinical progress. Although an accurate comparison of the ingested amount is difficult due to the lack of data in previous case reports, it seems likely that the patient in the present case had ingested a greater amount of the poisonous plant. In addition, the family of the patient who shared the same lunch showed abdominal pain, vomiting, and diarrhea, but the symptoms were mild and disappeared before the admission of the patient. The interview of the patient and her family confirmed that the patient had ingested most of the poisonous plant. Therefore, compared to the family as well as the patients in other cases, the patient in the present case is presumed to have shown more severe symptoms and clinical progress due to the ingestion of a far greater quantity of *C. palustris*.

To conclude, the acquisition of accurate toxicity data is not easy, especially when there is a paucity of studies on naturally-growing poisonous plants in a given area or an established system providing such data. Therefore, practical guidelines are often dependent on previously reported cases of suspected plant poisoning and its symptoms, treatment methods, and patient prognosis. However, the toxic compounds and their mechanisms of action are seldom precisely identified in many poisonous wild plants, and if there is a lack of sufficient case reports regarding the poisonous plant in question, as in the present case, the medical personnel treating the respective patient should consider that the patient may exhibit various side effects not included in previous reports or may have more severe clinical prognosis.

## Author contributions

**Conceptualization:** Won Young Sung.

**Supervision:** Won Young Sung.

**Writing – original draft:** Keun Taek Lee, Won Young Sung.

**Writing – review & editing:** Keun Taek Lee, Won Young Sung.

## References

[1] Kim DK Jang SB Choi YR Son DC Jung SY Oh SH. Field guide to poisonous plants of Korea: spring. 2021;Gyeonggi-do: Korea National Arboretum, p. 86–8.

[2] Park CW Ok TG Cho JH. The shock with bradycardia after ingestion of Caltha palustris. J Korean Soc Clin Toxicol 2004;2:41–4.

[3] Gummin DD Mowry JB Spyker DA Brooks DE Osterthaler KM Banner W. 2017 Annual report of the American Association of Poison Control Centers National Poison Data System (NPDS): 35th annual report. Clin Toxicol 2018;56:1213–415. 10.1080/15563650.2018.1533727 30576252

[4] Otter J Mayer S Tomaszewski CA. Swipe Right: a comparison of accuracy of plant identification apps for toxic pants. J Med Toxicol 2021;17:42–7. 32794048 10.1007/s13181-020-00803-6 PMC7785603

[5] Smit PG. A revision of Caltha (Ranunculaceae). Blumea - Biodiversity, Evolution Biogeography Plants 1973;21:121–50.

[6] Wink M. Mode of action and toxicology of plant toxins and poisonous plants. Julius-Kühn-Archiv 2010;421:93–112.

[7] Hill R VAN Heyninger R. Ranunculin: the precursor of the vesicant substance of the buttercup. Biochem J 1951;49:332–5. 14858339 10.1042/bj0490332 PMC1197509

[8] Plumlee K. Clinical Veterinary Toxicology. 2004;St Lous, MO: Mosby, p. 419.

[9] Umar SB DiBaise JK. Protein-losing enteropathy: case illustrations and clinical review. Am J gastroenterol 2010;105:43–9. 19789526 10.1038/ajg.2009.561

[10] Estrada O González-Guzmán JM Salazar-Bookman MM Cardozo A Lucena E Alvarado-Castillo CP. Hypotensive and bradycardic effects of quinovic acid glycosides from Aspidosperma fendleri in spontaneously hypertensive rats. Nat Prod Commun 2015;10:281–4. 25920261

[11] Jeon BH Kim CS Kim HS Park JB Nam KY Chang SJ. Effect of Korean red ginseng on blood pressure and nitric oxide production. Acta Pharmacol Sin 2000;21:1095–100. 11603282

[12] Hiwatashi K Shirakawa H Hori K. Reduction of blood pressure by soybean saponins, renin inhibitors from soybean, in spontaneously hypertensive rats. Biosci Biotechnol Biochem 2010;74:2310–2. 21071835 10.1271/bbb.100328

[13] Chen M Long Z Wang Y. Protective effects of saponin on a hypertension target organ in spontaneously hypertensive rats. Exp Ther Med 2013;5:429–32. 23404227 10.3892/etm.2012.856 PMC3570174

[14] Xu T Kuang T Du H. Magnoflorine: a review of its pharmacology, pharmacokinetics and toxicity. Pharmacol Res 2020;152:104632. 31911246 10.1016/j.phrs.2020.104632

